# Sleep to remember, sleep to protect: increased sleep spindle and theta activity predict fewer intrusive memories after analogue trauma

**DOI:** 10.1038/s41398-026-03910-0

**Published:** 2026-02-17

**Authors:** Yasmine Azza, Mathias K. Kammerer, Hong-Viet V. Ngo-Dehning, Mojgan Ehsanifard, Klaus Junghanns, Ines Wilhelm

**Affiliations:** 1 Faculty of Education, Free University of Bolzano-Bozen, Bolzano, Italy; 2 Department of Psychiatry and Psychotherapy, University of Lübeck, Lübeck, Germany; 3 Center of Brain, Behavior and Metabolism (CBBM), University of Lübeck, Lübeck, Germany; 4 Department of Psychology, University of Essex, Essex, UK

**Keywords:** Learning and memory, Human behaviour, Physiology, Predictive markers

## Abstract

Recent evidence shows a strong correlative link between sleep disturbances and intrusive memories after traumatic events, presumably due to insufficient (nocturnal) memory integration. However, the underlying mechanisms of this link and the role of specific neural activities during sleep are poorly understood so far. Here, we investigated how the intra-individual affective response to an experimental trauma predicts changes in oscillatory activity during subsequent sleep and how these changes predict the processing of the experimental trauma. In a randomized within-subject comparison, twenty-two female, healthy participants (23.14 ± 2.46 years) watched a well-validated film clip including “traumatic” contents and a neutral film clip before bedtime on two separate nights. Heart rate was recorded during the film clips and nocturnal brain activity was recorded using 64-channel high-density EEG during subsequent nights. Intrusive memories were assessed via a six-day diary and negative affect was assessed using experimental trauma film reminders one week after the trauma film. An increased intra-individual heart rate during the trauma film predicted higher intra-individual sleep spindle envelope the following night. Increased theta activity (4.25 - 8 Hz) during rapid eye movement (REM) sleep after the trauma film predicted fewer trauma film related intrusive memories and negative affect. Likewise, an increase in sleep spindles after the trauma film predicted fewer trauma film related intrusive memories. Our findings suggest that an experience-dependent up-regulation of these nocturnal oscillatory activity patterns, which are known to be involved in adaptive memory consolidation processes, serves as a protective factor against trauma-related intrusive memory development. Particularly, increased theta activity during REM sleep and sleep spindle activity seem to be of importance here.

## Introduction

Exposure to stressful or traumatic events, such as interpersonal violence, accidents, or natural disasters, is a significant risk factor for various psychopathologies, with posttraumatic stress disorder (PTSD) being the most frequent mental disorder that may develop after trauma. Intrusive memories – the involuntary re-experiencing of a traumatic event often triggered by situational reminders – can emerge in the aftermath of a traumatic event. Recurring and distressing intrusions are not only a hallmark symptom of PTSD but are also thought to be an early predictor of the development of PTSD or other psychopathological symptoms [1, 2]. According to well-recognized etiology models of PTSD (dual representation theory [3]; cognitive model of PTSD [1]; emotional processing theory [4]), intense arousal during the encoding of a traumatic event primarily stimulates perceptual and associative learning processes, which in turn can lead to an insufficient and fragmented memory trace of that experience. As a consequence, these unstructured memory representations may entail intrusive memories, which can be considered the result of a disruption in basic memory function [5].

Given this, gaining deeper insights into mechanisms underlying these impaired memory functions after traumatic experiences is crucial. One key factor in memory consolidation is sleep. Neural activity during sleep plays a vital role in integrating new experiences into existing memory systems [6] and in diminishing the emotional intensity of distressing memories [7]. Thus, it is not surprising that previous studies were able to show that sleep in the early aftermath of an analogue trauma can help reduce intrusive experiences and intrusion-related distress [8–11]. Furthermore, decades of sleep science provide extensive evidence that slow-wave sleep activity, sleep spindle activity (brief bursts of neural activity during non-REM sleep (NREM)), and theta activity during rapid eye movement (REM) sleep are pivotal for successful memory consolidation. Additionally, most oscillatory activity during REM sleep can be attributed to the limbic system, a brain region known to be crucial for emotional memory consolidation [12, 13]. However, there is growing evidence that neural activities during NREM and REM sleep complement each other in preserving and solidifying declarative aspects of an emotional memory, while at the same time attenuating its affective charge [14, 15]. With regard to intrusive memories that emerge after traumatic experiences, these adaptive processes seem to fail. This seems particularly relevant, as individuals with PTSD often exhibit pronounced deficits in NREM and REM sleep [16, 17], which may not only contribute to the initial development of symptoms following trauma but also potentially act as a maintaining factor by impairing further adaptive emotional processing. Thus, understanding the underlying mechanisms and roles of specific sleep oscillations that promote adaptive memory consolidation of traumatic experiences, and thereby potentially protect against intrusion development seems paramount in order to bring forth early and late interventions.

One way to investigate these underlying mechanisms is the use of an analogue trauma film paradigm. In this paradigm, highly distressing film content (e.g., interpersonal violence) is presented and elicited responses – which have been shown to be analogous to symptoms experienced after actual trauma (e.g., intrusive memories, physiological arousal, negative mood) – can be measured [18, 19]. Experimental sleep studies that used such trauma film paradigms have shown that increased slow-wave activity during a nap after film presentation was associated with less intrusion-related distress during the following week [10]. Additionally, increased slow-wave activity has been observed following stressful experiences, possibly reflecting enhanced post-event processing [20, 21]. Furthermore, it has been shown that increased sleep spindle activity after film presentation was related to fewer intrusive memories [8], to adaptive emotional memory processing [22], and to improved sleep-dependent anxiety regulation [23]. Furthermore, a daytime nap *with* REM sleep (as opposed to without) after the exposure to an analogue trauma film entailed fewer subsequent intrusions [10] and decreased the aversiveness of intrusive memories [24] in healthy individuals. Additionally, in a recent between-subject design study, greater theta activity during REM sleep after an experimental trauma was associated with significantly less intrusive memories [25]. Further supporting the potential protective effects of theta activity, a cross-sectional study showed that individuals who experienced a traumatic event but did not develop PTSD, exhibited higher theta activity compared to individuals who developed PTSD [26]. However, these links need replication, have in parts not yet been studied in an overnight sleep study, and – most importantly – need to be investigated in a randomized within-subject design to inform about potential causality and inter-individual protective factors. Understanding causality and potential protective factors may help refine interventions to enhance sleep oscillatory activity – such as auditory closed-loop stimulation [27] – and ultimately improve trauma-related memory processing in the early aftermath of trauma, thereby reducing the risk of PTSD development.

Given that only 10–20% of the people who experienced a trauma develop clinical post-traumatic symptomatology [28], this study aimed to determine potential protective factors against intrusive memory formation in a randomized within-subjects design in healthy individuals. For that, we used a validated, highly distressing film clip (i.e., “trauma” film) and a neutral film clip to examine how intra-individual changes in sleep physiology might protect from intrusion development. Firstly, we explored (i) the links between peri-trauma-film heart rate (as an indicator for arousal during encoding) and subsequent sleep oscillatory activity. Secondly, we expected (ii) increased EEG oscillatory activity in the slow wave, theta, and spindle spectrum after the trauma film exposure. Finally, we expected (iii) increased slow-wave activity, sleep spindle activity, and theta activity to be predictive of fewer trauma film-related intrusive memories and negative affect.

## Methods

### Participants

An a priori power analysis was conducted using G*Power 3.1 (one-tailed test, α = 0.05). Effect sizes reported in previous studies (r = 0.47 – 0.56) were used as reference values, resulting in an estimated optimal sample size ranging from n = 18 to n = 26 to achieve a statistical power of 0.80. Twenty-two healthy and female participants (mean age: 23.14 ± 2.46) took part in the present study and were recruited by the student mailing list of the University of Lübeck, Germany. Prior to the experiment the Ethics Commission of the University of Lübeck approved all study protocols and participants provided written informed consent. As compensation, participants could choose to either receive a monetary incentive or course credits for their study program. Exclusion criteria were assessed in advance via a semi-structured telephone interview. Specifically, participants were asked whether they had ever experienced interpersonal trauma (e.g., sexual or physical assault) via a binary yes/no question. In addition, a standardized checklist of potentially traumatic and stressful life events was administered, based on the German version of the Traumatic Life Events Questionnaire (TLEQ; [29]). This included 16 specific life events (e.g., serious illness or accident involving a family member, death of a close person, traumatic incident at work), for which participants indicated whether they had experienced the event, how distressing it had been, and when it occurred. Participants were excluded if they endorsed any event as having occurred and rated it as highly distressing. The full exclusion criteria were: (1) experience of traumatic events involving interpersonal violence, (2) highly distressing life events reported on the TLEQ, (3) frequent viewing of violent films, (4) presence of a neurological or psychiatric disorder, (5) reported habitual consumption of alcohol or cannabis, (6) scheduled intake of medication influencing sleep during the study interval, and (7) shift work.

### Procedure

In a randomized within-subject comparison, each participant spent three nights in the sleep laboratory including polysomnographic recordings. A standardized protocol was used to prevent participants from engaging in any individual activities and to minimize variability in the time interval between trauma film exposure and sleep (20 min maximum). Participants entered the laboratory at individually scheduled times, closely aligned with their habitual bedtimes, to control for circadian influences. Notably, bedtime and wake-up times were standardized within participants across nights to ensure comparability of sleep time opportunity.

**Fig. 1:**
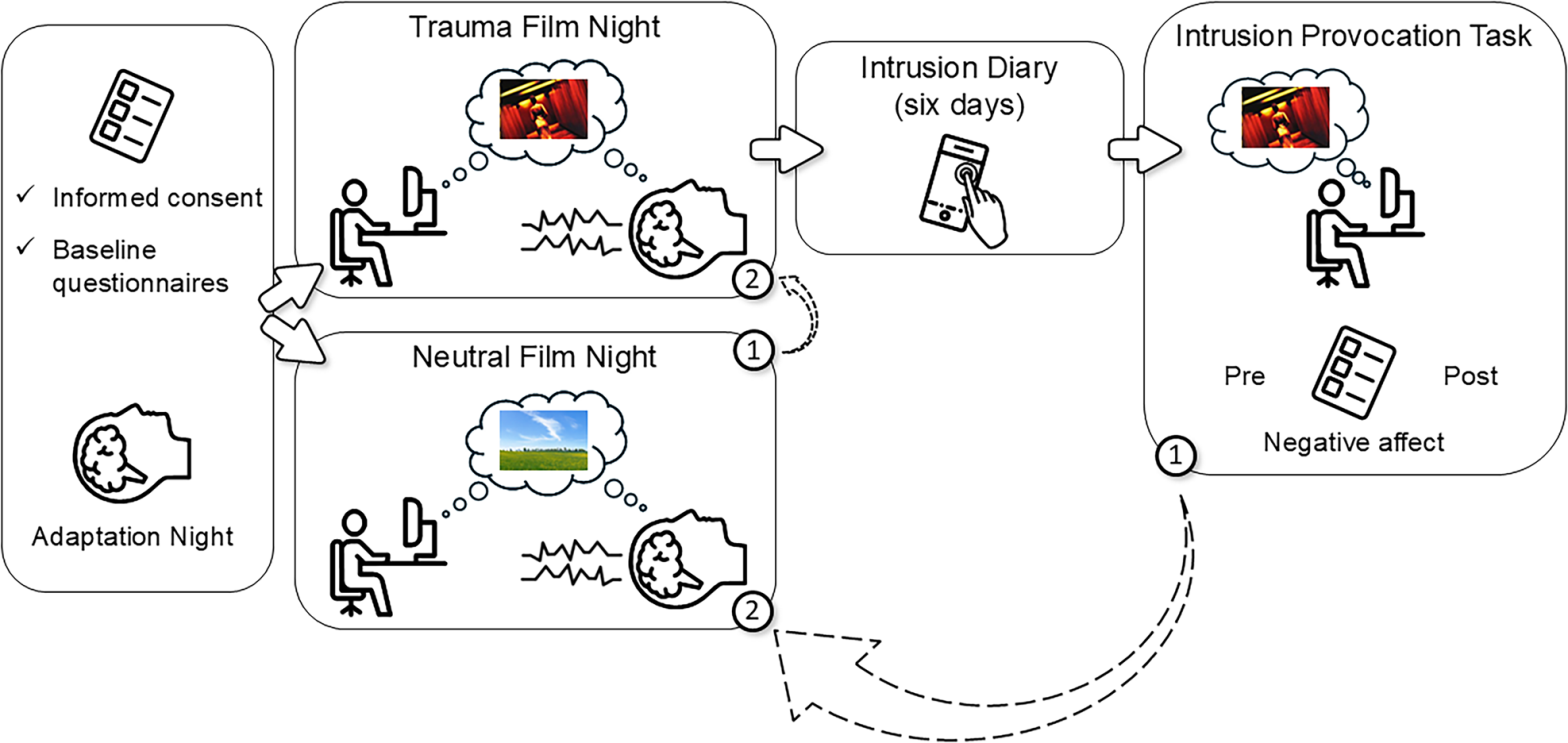
Study design. In a randomized within-subject comparison, participants watched either first a film clip including distressing contents (“Trauma Film”) or a neutral film clip (“Neutral Film”) before bedtime with polysomnography. All participants underwent both conditions on two separate nights (test-nights). For both film clips, heart rate and subjective arousal and mood were assessed. Intrusion diary and intrusion provocation task were only administered after trauma film night. There was a minimal time gap of two days between adaptation night and first test-night and a minimal time gap of seven days between each test-night. Numbers (1) and (2) indicate in what sequence an individual underwent the study procedure depending on randomized assignments.

Participants were assigned to the experimental conditions based on a predefined randomization list. The sequence was generated prior to the study and participants were allocated to conditions in the order of their arrival. The first night (T0) served as an adaptation of sleep to the new environment (i.e., adaptation night). Before going to sleep, participants filled in informed consent and a baseline battery of questionnaires including demographic data, the WHO-Five Well-Being Index [30], the Emotion Regulation Questionnaire [31], the Pittsburgh Sleep Quality Index [32], and the Edinburgh Handedness Inventory [33]. On the second night (T1), according to a predefined randomization list, participants either watched a neutral or a highly distressing 12-minute film clip (see section “Trauma film paradigm”) in a darkened room using headphones. They were further informed that the film material they will see could contain violent and/or distressing scenes and that they are free to withdraw from the experiment at any point in time. The third night (T2) took place with a minimal time gap of seven days. It was conducted identically to T1 except that participants watched the film clip they had not been presented with before (neutral or trauma film). After the trauma film night, participants were instructed to document every intrusive memory that came to mind over the following six days via an online questionnaire linked on their smartphone (i.e., intrusion diary). Additionally, participants were exposed to trauma film reminders seven days after the trauma film night in our laboratory and were asked to rate their subjective negative affect before and after the presentation of trauma film reminders (i.e., intrusion provocation task). All participants underwent both conditions on two separate nights (see also Fig. 1).The following definition of what constitutes an intrusion was given for the intrusion diary and the intrusion provocation task: “Intrusions are memories of a highly distressing situation that come to mind spontaneously and involuntarily, in the form of images, thoughts, or sounds. They are usually triggered by a cue. A cue is something that is linked to the original emotional memory (i.e., something that reminds of the film content).”.

### Trauma film paradigm

The analogue traumatic stimulus in this study consisted of a previously used and validated 12-minute scene from the movie “Irreversible”, directed by Gaspar Noé including scenes of sexual violence (e.g., [8, 34]). Physiological and subjective reactions to the stimulus material were compared to a 12-minute neutral film depicting neutral social interactions as well as architectural scenes. Heart rate was recorded during both film presentations using the same physiological setup as during sleep, via bipolar ECG electrodes (left lower rib cage and right clavicle) and a BrainAmp amplifier system (BrainProducts GmbH) at a sampling rate of 500 Hz. Before and after both films, participants rated their mood and levels of arousal on a visual analogue scale, namely the Self-Assessment Manikins (SAM) [35].^1^

### Sleep EEG recordings and analyses

Brain activity during sleep was recorded using a Brain Vision system (LiveAmp) with a 64-channel electrode cap (BrainCap) and BrainAmp amplifiers (Brain Products, Munich, Germany). Two vertical electrooculogram (EOG) electrodes were placed above and below the left eye and two additional ones next to the lateral canthi to record horizontal eye movements. Three electromyography (EMG) electrodes (left and right upper chin and beneath chin) and two electrocardiography (ECG; left lower rib cage and right clavicle) electrodes were additionally applied. Impedances were kept below 10 kΩ. Brain activity was recorded with a sampling rate of 500 Hz.

All EEG analyses were performed using Matlab 2016b [36] and the Fieldtrip toolbox [37]. First, all recordings were re-referenced to the mean activity of both mastoids and sleep stages were determined by visual scoring according to the American Academy of Sleep Medicine standard criteria (AASM; [38]) using the software SchlafAus 1.0 (developed by Steffen Gais, unpublished, University of Tuebingen, Germany). Data was further preprocessed by inspecting all channels of each person for possible distortions and marking them. All data was then high-pass filtered at 0.1 Hz and beforehand defined bad channels were interpolated. Following this, the filtered signals were segmented separately for NREM (i.e., N2 and N3) and REM sleep. For each participant and condition, artifact-free epochs of 8.192-s were extracted using a 50% overlap and a Hanning window. A Fast Fourier Transform (*mtmfft* function in Fieldtrip) was then applied, and the resulting power spectra were averaged across all segments. This yielded power spectra up to 30 Hz with a frequency resolution of 0.122 Hz. Individual slow wave activity (SWA; 0.5 – 4 Hz) during NREM and theta power during REM (4.25 – 8 Hz) were determined by averaging the spectral power across the respective frequency bins for each participant, condition, and electrode. Spatial patterns were visualised with topographical maps depicting the SWA or theta power at each electrode averaged across all participants for each condition. Moreover, differences between conditions were illustrated using topographical maps, which were generated by subtracting SWA or theta power obtained after the neutral film from the trauma film condition at each electrode, and then averaging across all participants. Discrete sleep spindle events (12 – 16 Hz) were detected during artifact-free intervals of NREM sleep 2 and 3 as described in [39]. For further information on sleep spindle detection and quantification of intra-individual increase in slow-wave activity, see Supplementary Material M2.

### Intrusion diary

Intrusive memories were assessed via a mobile daily for six days following the traumatic film clip via a questionnaire link installed on the participant’s smartphone. The time and date of the entries were recorded. Participants were instructed to exclude deliberate or intentional recollections (e.g., consciously thinking or talking about the film). Each entry included a control item on suddenness, allowing a differentiation between spontaneous intrusions and voluntary memories. Both cue-triggered and uncued spontaneous intrusions were documented only if they were clearly related to the film. Participants indicated the sensory form (image, sound, or thought) and rated associated arousal and distress on a scale from 0 = *not at all* to 100 = *very distressing/aroused*. (adapted from [40]). To ensure accuracy, a short debriefing interview was conducted after the six-day period to clarify ambiguous entries and confirm completeness; only memories meeting the intrusion definition were retained. Participants received daily email reminders in the evening to remind them of entering possible memories in case they forgot. The main outcome variable was defined as the total number of intrusions reported over the six-day period after trauma film exposure, which is a commonly used measure [8–10]. For further information, see Supplementary Material M1.

### Intrusion provocation task

During an intrusion provocation task seven days after the trauma film presentation, five trauma-film-associated pictures (i.e., trauma film reminders) were presented for four seconds each (suit, dark street, stairs, underpass, and a pedestrian tunnel). These pictures were not extracted from the film itself but were thematically related and served as reminders of its content. After each of the pictures, during a one-minute interval, participants were asked to place their fingers on the spacebar, close their eyes, and press the spacebar for every upcoming intrusion. The total number of indicated intrusions for each image was calculated. Before and after the task, self-reported negative affect was evaluated using the Positive and Negative Affect Schedule - German Version [41].

### Statistical analysis

Descriptive statistics and comparisons of mean values were analyzed using R [42]. To validate that the trauma film induced an increase in arousal and negative mood in our sample, paired t-tests (pre/post film presentation) for each of the conditions (trauma/neutral) were calculated. To examine potential differences in topographical activity patterns between the two conditions, a non-parametric cluster-based permutation testing was conducted separately for the frequency bands of slow-wave activity (0.5 – 4 Hz), theta activity (4.25 – 8 Hz), and spindle activity (12 – 16 Hz) [43]. These tests were implemented in FieldTrip [37]. To this end, electrodes were selected that showed significant differences in between conditions (two-tailed paired-samples t tests, sample-level alpha = 0.05). In the resulting statistical map, adjacent samples were grouped into positive and negative clusters for which cluster-level statistics were calculated by summing up the t-values within each cluster. These were tested against a reference distribution (cluster-level alpha = 0.05), generated by shuffling the association of data and condition (1000 permutations) and, for each permutation, taking the maximum statistics among all clusters.

To examine individual-level associations between physiological reactivity and sleep architecture, intra-individual change scores (trauma vs. neutral condition) were computed for heart rate and sleep measures. To further characterize physiological reactivity, we analyzed heart rate minute-by-minute across both film conditions and performed paired t-tests at each time point. This data-driven approach revealed that heart rate differed most strongly between the trauma and neutral film during minutes seven to nine, corresponding to the most distressing segment of the trauma film (all *p* < 0.01). Accordingly, the HR outcome variable used in subsequent analyses was defined as the mean heart rate across minutes seven to nine. This allowed us to focus on the peak emotional response period while minimizing noise from non-specific anticipatory arousal present at earlier time points. Spearman correlation analyses were then used to test whether changes in heart rate during film viewing predicted subsequent changes in spatial patterns of sleep oscillatory activity. Statistical assessment was based on the same non-paramentric cluster-based permutation approach, as described above.

Finally, we investigated whether spatial intra-individual changes in sleep measures (i.e., slow wave activity, sleep spindle activity, theta activity) following trauma exposure predicted behavioral outcomes − namely, the number of intrusive memories recorded in the six-day diary and negative affect during the intrusion provocation task. Again, following a Spearman correlation, we applied non-parametric cluster-based permutation tests to assess statistical differences. Because four correlations were conducted per behavioral outcome, we applied a false discovery rate (FDR) correction using the Storey procedure resulting in adjusted *q*-values [44]. To further characterize significant effects, average spectral power across electrodes, which contributed to each significant cluster, was extracted and correlated with the behavioral outcome using Spearman’s ρ. Confidence intervals (95%) for the correlation coefficients were estimated via bootstrapping with 1000 iterations using the correlation package in R.

All correlation tests were one-sided, based on a-priori directional hypotheses derived from previous research indicating that increases in specific oscillatory activities (e.g., spindle activity, theta activity) are associated with more adaptive emotional memory processing.

## Results

### Effects of experimental trauma film on arousal, mood, and sleep architecture

In line with previous studies, the participants’ moods significantly deteriorated (t(21) = 5.78, p < 0.0001, *M* = 2.55, 95% CI [1.63, 3.46]), while arousal levels significantly increased (*t*(21) = −4.64, *p* < 0.001, *M* = −2.00, 95% CI [−2.90, −1.10]) by the presentation of the trauma film. For the neutral film, mood scores did not change significantly, whereas arousal levels significantly decreased (*t*(20) = 2.74, *p* = 0.013, *M* = 1.00, 95% CI [0.24, 1.76]) from pre to post (see also Supplementary Material Table T1).

In addition to these within-condition effects, we also compared the pre–post change scores (post minus pre) between conditions using paired-sample t-tests. These analyses confirmed that mood deteriorated more significantly following the trauma film compared to the neutral film, t(20) = –5.51, *p* < 0.001, *M* = –2.62, 95% CI [–3.61, –1.63]. Likewise, arousal increased more significantly after the trauma film than the neutral film, t(20) = 6.05, *p* < 0.001, *M* = 3.10, 95% CI [2.03, 4.16].

Consistent with the emotionally distressing content of the trauma film, heart rate was significantly elevated compared to the neutral film during specific segments. As described in the Methods section, minute-by-minute comparisons revealed the strongest differences during minutes seven to nine, resembling the most intense segment of the trauma film (all *p* < 0.01; see Supplementary Figure S4). These findings indicate a transient but robust physiological response to the trauma content.

We further examined whether there were any detectable differences in sleep architecture when comparing the trauma with the neutral film condition. Participants showed significantly increased sleep onset latencies (*t* = 2.73, df = 21, *p* = 0.013) after the trauma film while there was no difference between conditions in any other sleep variable (see Table 1). Interestingly, participants showed a flatter slope of SWA increase during the first sleep cycle across 41 channels primarily over the right hemisphere after the trauma film condition exposure (*t* = −3.54 – −2.08, df = 21, *p* ≤ 0.01). See also Supplementary Figure S2 for a depiction of differences in oscillatory activity between conditions.

**Table 1:** Sleep architecture and oscillatory activity after trauma and neutral condition.

	Trauma film Mean ± SEM	Neutral film Mean ± SEM	*t*(21)	*p*
Sleep onset latency (minutes)	**23.69** ± **2.79**	**18.15** ± **1.72**	**2.73**	**0.013**
NREM 1 (%)^a^	4.3 ± 0.55	3.93 ± 0.38	0.84	0.412
NREM 2 (%)^a^	55.98 ± 1.12	56.88 ± 1.42	−0.61	0.547
NREM 3 (%)^a^	13.37 ± 0.8	13.85 ± 0.96	−0.58	0.570
REM sleep (%)^a^	21.24 ± 0.87	20.89 ± 0.89	0.56	0.579
Total sleep time (minutes)	474.59 ± 8.35	473.98 ± 9.13	0.89	0.891
Wake after sleep onset (%)	5.09 ± 1.14	4.42 ± 0.92	0.56	0.579
Slow-wave activity	19.94 ± 1.54	19.98 ± 1.68	–0.07	0.942
SWA slope (first sleep cycle)	**2.36** ± **0.19**	**3.00** ± **0.29**	**–3.71**	**0.001**
Spindle count	1845.81 ± 80.37	1855.08 ± 77.22	–0.13	0.900
Spindle density	4.91 ± 0.12	4.92 ± 0.15	–0.03	0.975
Spindle envelope	6.58 ± 0.27	6.58 ± 0.28	0.01	0.995
REM theta power	0.62 ± 0.05	0.60 ± 0.05	0.87	0.395

^a^Proportion of sleep stages relative to total sleep time.

*SEM* standard error of the mean, *REM* rapid eye movement sleep, *NREM* non-REM sleep stages, *SWA* slow wave activity. Bold values indicate significant effects with *p* < 0.05.

### Descriptions of reported intrusions

Following exposure to the trauma film, participants reported an average of 2.24 intrusive memories (SD = 1.45) over the six-day monitoring period. Only entries that were classified as sudden and clearly related to the trauma film content were included. Example diary entries illustrate the nature of these experiences. One participant wrote: “I heard things the man in the film said to the woman – insults, isolated words,” reflecting an auditory intrusion. Another participant described a visual intrusion: “I saw some images – red walls – but not the person from the film, while walking through an underpass,” indicating a context-triggered, scene-related memory. While individual differences in intrusion frequency were apparent, a general decline over the six-day period was observed descriptively. The distribution and temporal trajectory of intrusions across the six days are displayed in Supplementary Figure S1.

### Effects of experimental trauma film exposure on neural sleep correlates

Average slow-wave activity (*t* = −2.15 – 1.87, *p* > 0.05) and sleep spindle activity (count: *t* = −1.45 – 1.42, *p* > 0.05; envelope: *t* = −1.12 – 1.01, *p* > 0.05) during NREM sleep, as well as average theta activity (*t* = −1.41 – 1.99, *p* > 0.05) during REM sleep after trauma film exposure did not differ significantly compared to that after the neutral film (see also Supplementary Figures S2 and S3).

### Correlation between peri-trauma-film heart rate and sleep oscillations

**Fig. 2:**
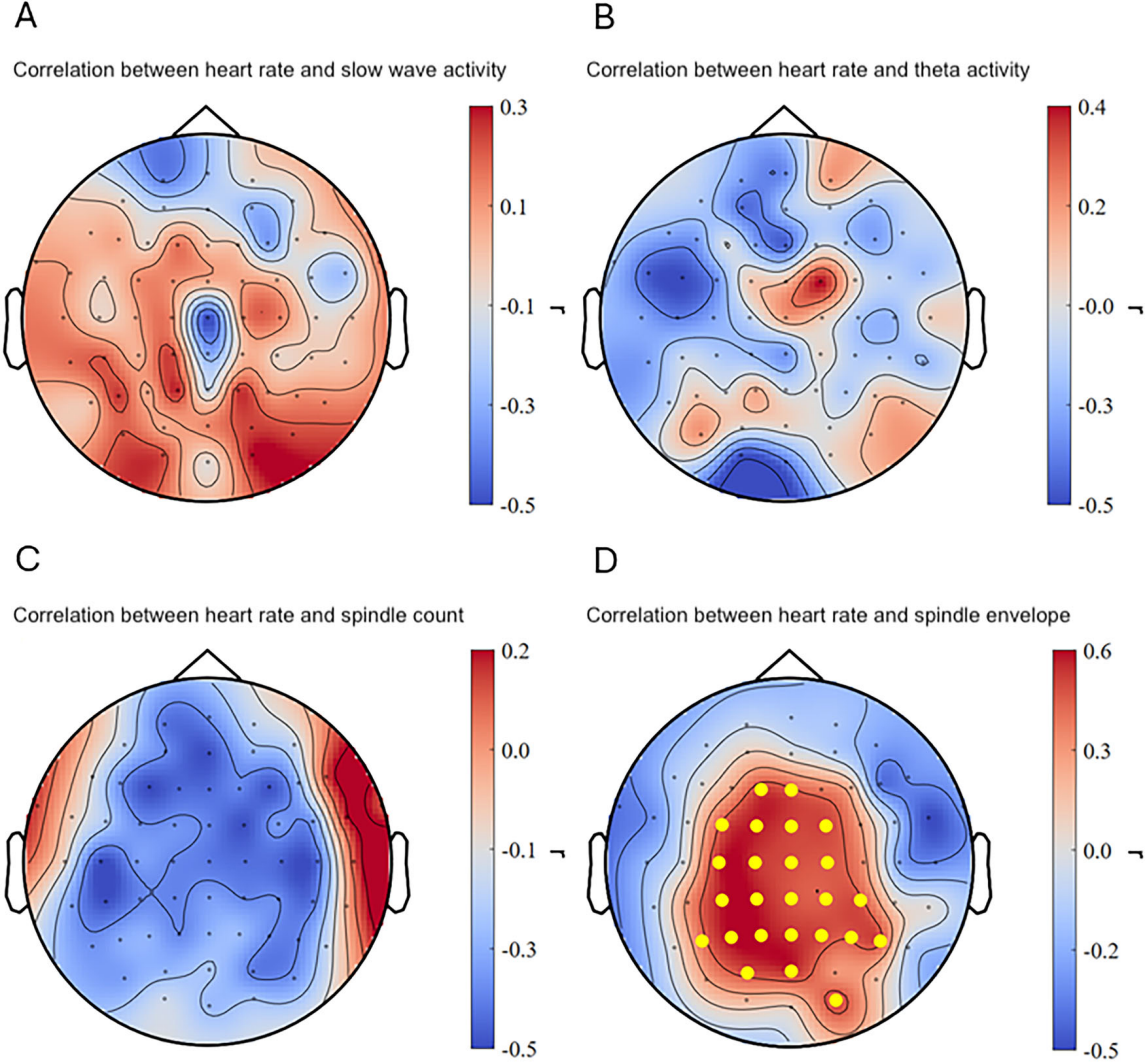
Correlation between heart rate during film exposure and sleep measures. (**A**–**C**) No significant correlations were found for slow-wave activity, theta activity, and sleep spindle count. (**D**) Intra-individual increase in heart rate during the trauma film (as compared to the neutral film) significantly predicted intra-individual increase in sleep spindle envelope after the trauma film (compared to neutral). Dots in yellow mark the channels contributing to the significant cluster.

Individual peri-trauma-film heart rate during the trauma film did not significantly predict slow-wave activity (see Fig. 2A) or theta activity the following night (see Fig. 2B). Intra-individually increased peri-trauma-film heart rate significantly predicted intra-individually increased sleep spindle envelope the following night in 25 channels over frontal, central, parietal, and parieto-occipital areas (cluster-based Spearman’s ρ = 0.556, *p* < 0.05, *q* = 0.040, 95% CI [0.057, 0.874] (range of individual Spearman’s ρ = 0.586 – 0.404); see also Fig. 2D). No significant correlation was found for sleep spindle count (see Fig. 2C).

### Prediction of intrusions and affective response to trauma film reminders

**Fig. 3:**
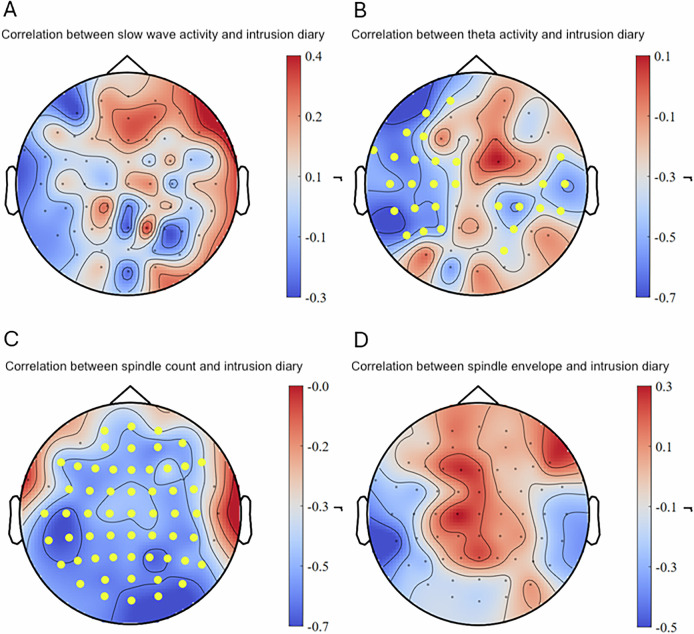
Correlations between intra-individual sleep changes and intrusions. An intra-individual increase of theta activity (**B**) and sleep spindle count (**C**) after the trauma film (compared to neutral) was significantly associated with less intrusive memories reported during the six-day intrusion diary period. No significant correlations were found for slow-wave activity and sleep spindle envelope (**A** and **D**). Dots in yellow mark the channels contributing to the significant cluster.

**Fig. 4:**
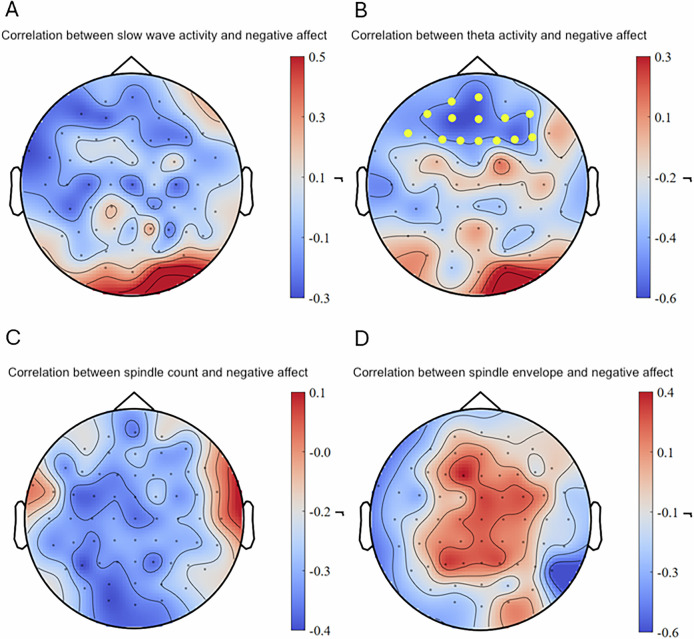
Correlations between intra-individual sleep changes and negative affect. An intra-individual increase of theta activity (**B**) after the trauma film (compared to neutral) was significantly associated with less negative affect during the intrusion provocation task one week after the trauma film exposure. No significant correlations were found for slow-wave activity, sleep spindle count and sleep spindle envelope (**A,** **C**, and **D**). Dots in yellow mark the channels contributing to the significant cluster.

Individual changes in slow-wave activity after the trauma film exposure did not predict intrusive memory formation during the six days intrusion diary period nor did it predict negative affect during the intrusion provocation task (see Figs. 3A and 4A). As hypothesized, an intra-individual increase in theta activity during REM sleep over left frontal, temporal, and parietal as well as right temporal and parietal areas in 30 channels after the trauma film significantly predicted less intrusive memories during the six days intrusion diary period (cluster-based Spearman’s ρ = −0.62, *p* < 0.05, *q* = 0.038, 95% CI [−0.879, −0.236] (range of individual Spearman’s ρ = −0.747 – −0.364); see also Fig. 3B). Similarly, intra-individually increased theta activity during REM sleep over frontal areas in 14 channels after the trauma film predicted less negative affect during the intrusion provocation task (cluster-based Spearman’s ρ = −0.551, *p* < 0.05, *q* = 0.049, 95% CI [−0.842, −0.088] (range of individual Spearman’s ρ = −0.592 – −0.384); see also Fig. 4B). Intra-individually increased sleep spindle counts across nearly the entire scalp in 56 channels after the trauma film predicted significantly fewer intrusive memories during the six days intrusion diary period (cluster-based Spearman’s ρ = −0.590, *p* < 0.01, *q* = 0.025, 95% CI [−0.813, −0.185] (range of individual Spearman’s ρ = −0.672 – −0.366); see also Fig. 3C). Other individual changes in sleep spindle activity after the trauma film did not predict intrusive memories nor negative affect during the intrusion provocation task (see Figs. 3D, 4C, D). For results on sleep spindle density, see Supplementary Figure S5.

## Discussion

Here, we differentiated for the first time the impact of an experimental trauma film versus a neutral film on intra-individual neural sleep activity using high-density EEG recordings in a within-subjects design. This allowed for conducting fine-grained cluster analyses of associations between intra-individual changes in sleep physiology and the processing of aversive experiences.

Against our hypothesis, we did not find significant changes in mean slow-wave activity, sleep spindle activity, and theta activity after the trauma film compared to the neutral film. This most likely can be attributed to high inter-individual variability in our sample in response to a comparatively “mild” stressor. Since this study comprised only healthy participants, it is likely that they responded very differently to this “mild” stressor on an individual level. Consequently, the effects of the stressor may be present at the individual level but not on average across participants.

However, in line with our expectations and with previous studies [10, 25, 26], intra-individually increased theta activity during REM sleep after the trauma film predicted less intrusive memories in the intrusion diary and less negative affect in the intrusion provocation task. This strengthens the notion of theta activity being an affective “depotentiator” [45] and being actively involved in adaptive emotional memory consolidation [46]. Congruently, previous studies have shown that increased REM theta power significantly predicted enhanced emotional memory consolidation after a stress exposure [47]. Thus, our findings support the notion that increased theta activity during REM sleep after a traumatic event is a protective factor against intrusive memories and trauma-related negative affect. Moreover, our study is the first to demonstrate that intra-individual changes in theta activity are associated with fewer intrusions, suggesting that experience-dependent modulation of theta activity may serve an adaptive function following (analogue) trauma exposure.

Furthermore, in accordance with previous studies [8, 10], we found an intra-individually increased sleep spindle count after the trauma film to predict less intrusive memories in the intrusion diary the following week. This aligns with research that showed increased sleep spindle activity to be linked to reduced sleep-dependent anxiety responses in traumatized individuals [23]. Additionally, as sleep spindles change in response to experiences and promote synaptic plasticity [48], they play a key role in flexibly adapting to new demands. In sum, our findings suggest that increased sleep spindle activity facilitates adaptive emotional memory consolidation after traumatic events, helping to mitigate the development of intrusive traumatic memories [49]. However, individuals with PTSD often show increased sleep spindles, which has been suggested to reflect maladaptive over-consolidation of traumatic events and thus lead to more intrusive memories [50]. Considering these somewhat opposing findings, a differentiated assessment seems necessary. In light of the correlation between intra-individually increased heart rate during trauma film exposure and subsequent increased sleep spindle envelope in this study, we propose temporarily increased sleep spindle activity after an acute stressor (accompanied by temporarily increased arousal) to reflect adaptive emotional memory processing. In contrast, increased sleep spindle activity in PTSD patients (accompanied by chronic hyperarousal) could reflect some sort of “dysfunctional replay” as a maladaptive attempt of the sleeping brain to integrate traumatic experiences into existing memory systems (see also [49]).

Thus, one could speculate that moderately increased emotional reactivity (i.e., elevated heart rate) during a stressful event promotes adaptive nocturnal emotional processing by up-regulating sleep spindle activity, which may mitigate intrusion development. Corroborating this, previous studies found that reduced heart rate during a trauma film predicted more frequent intrusive images in healthy individuals (e.g., [51]) and that attenuated skin conductance responses (i.e., physiological arousal) during an emotion regulation task predicted later increased intrusion development in trauma-exposed individuals [52]. This is further supported by an affect-specific link between heart rate and neural activity in integral parts of the limbic system [53, 54], a network crucial for memory consolidation [55] and known to be altered in individuals with PTSD [56]. These findings suggest a broader mechanism involving the autonomic nervous system and its interaction with limbic structures such as the amygdala, hippocampus, and prefrontal cortex [55]. Sympathetic activation during emotional events may facilitate memory encoding by enhancing amygdala-hippocampal interactions and increasing stimulus salience [57]. Additionally, experience-dependent fluctuations in autonomic activity during sleep are linked to certain sleep oscillatory dynamics and emotional memory processing [58–61]. Taken together, these findings underscore the importance of a balanced autonomic response not only during the traumatic experience itself, but also during subsequent sleep, where it may support emotional memory processing. In line with this, emotional engagement in the context of traumatic events – up to a certain degree and as opposed to detachment (i.e., dissociation) – has been shown to be beneficial in preventing or reducing PTSD symptoms, presumably by supporting adaptive (nocturnal) emotional memory processing [62, 63]. However, it remains unclear whether specific levels or timing of arousal around a traumatic event benefit or hinder adaptive memory encoding and subsequent (nocturnal) consolidation, with the role of autonomic activity during sleep in modulating emotional memory processing and oscillatory activity still poorly understood (see e.g., [51] for an attempt to decipher distinct effects of increased arousal at different time points around an analog traumatic event).

Strengths of our study include the conduct of a within-subjects design, which allowed us to assess intra-individual changes in heart rate and sleep measures, thereby emulate real life inter-individual differences in response to traumatic events more accurately and ultimately allowed us to examine how this variability predicted the development of intrusive memories. This analytical approach is based on the rationale that intra-individual variability in sleep physiology may provide more meaningful insight into psychological outcomes than group-level mean differences. Furthermore, instead of daytime naps, we used whole night high-density EEG recordings, which warrant higher spatial resolution and independence from ultradian phases. As to limitations, our study comprised a relatively small sample of only female, non-clinical participants, which limits generalizability. Nonetheless, a post-hoc power analysis indicated that the study was sufficiently powered to detect medium to large effects, but likely underpowered to reliably detect small to medium associations. Thus, non-significant findings should be interpreted with caution. Further, although analogue trauma film paradigms are widely used, they do not necessarily reflect real life trauma and our findings therefore would ideally need replication in naturalistic settings. Also, during the six days intrusion diary period, participants received email reminders every evening to retrospectively report any missed intrusions, which may have introduced some reporting bias. Additionally, as sleep data were only collected for single nights, future studies should investigate how sleep oscillations evolve over time following trauma exposure and how they relate to the development of intrusions.

In conclusion, our findings provide further evidence that theta activity during REM sleep and sleep spindle activity during NREM sleep after analogue trauma are modulated in an experience-dependent manner and, by this, being protective against intrusion development. Furthermore, our findings suggest a close interplay between physiological reactivity during a traumatic event and subsequent sleep spindle activity. Given that sleep disturbances are prevalent in individuals with PTSD [64] and are likely to contribute to PTSD symptom formation in the aftermath of traumatic events [65], interventions that aim to stabilize post-traumatic sleep quality seem paramount. Additionally, according to our findings, improving theta and sleep spindle activity after a traumatic event could promote adaptive emotional memory consolidation and thereby possibly prevent PTSD symptom formation (for theoretical reviews, see [66, 67]).

## Supplementary information

Supplementary Material

Supplement Figure S1

Supplement Figure S2

Supplement Figure S3

Supplement Figure S4

Supplement Figure S5

## Data Availability

All Matlab codes and functions used for data analysis as well as behavioral data are publicly available on a GitHub repository: https://github.com/ngohv/Azza_TranslPsychiatry_2026. EEG and ECG Data can be made available upon request.

## Footnotes

1 We acknowledge that watching a movie scene with distressing contents does not necessarily reflect witnessing real life trauma. We use the term “trauma film” for reasons of better readability only
