# Supplementary material for: Sleep to remember, sleep to protect: increased sleep spindle and theta activity predict fewer intrusive memories after analogue trauma
Source: Transl Psychiatry. 2026 Feb 17;16:147. doi: 10.1038/s41398-026-03910-0 (PMC12987997; doi:10.1038/s41398-026-03910-0)
Supplement: Supplementary file 1 — Supplementary Material [file 41398_2026_3910_MOESM1_ESM.docx]

**Corresponding to Results: Effects of experimental trauma film on arousal, mood, and sleep architecture**

**Table T1. Mood and arousal in response to the film material**

|  | Pre Film  Mean ± SEM | Post Film  Mean ± SEM | *t(df)* | *p* |
| --- | --- | --- | --- | --- |
| Trauma Film |  |  |  |  |
| Mood (SAM) | **6.55±0.34** | **4±0.33** | **5.78(21)** | **<.0001** |
| Arousal (SAM) | **4±0.43** | **6±0.34** | **-4.64(21)** | **<.0001** |
| Neutral Film |  |  |  |  |
| Mood (SAM) | 6.9±0.32 | 6.86±0.28 | 0.22(20) | .825 |
| Arousal (SAM) | **3.57±0.51** | **2.57±0.40** | **2.74(20)** | **.013** |

*Note.* SEM = standard error of the mean; SAM = Self-Assessment Manikin

**Corresponding to Results: Descriptions of reported intrusions**

**Figure S1. Distribution and temporal trajectory of intrusions across the six days of intrusion diary period**

*Note.* Boxplots show the frequency distribution of intrusions per day averaged across participants.

**Corresponding to Results: Effects of experimental trauma film exposure on neural sleep correlates**

**Figure S2. Boxplots of sleep parameters by experimental condition**

*Note.* Boxplots display the distribution of six sleep-related variables for the trauma and neutral film conditions (each n=22). Each box represents the interquartile range (25th–75th percentile), the horizontal line within the box indicates the median, whiskers reflect 1.5 times the IQR, and outliers are shown as individual points.

**Corresponding to Results: Effects of experimental trauma film exposure on neural sleep correlates**

**Figure S3. Differences in frequency bands comparing trauma and neutral film**

*Note.* Topographical distribution of the mean power spectra in three parameters of interest (slow-wave activity (A): 0.5 - 4Hz; theta activity (B): 4.25 - 8Hz; spindle count (C), and -envelope (D): 12 - 16Hz) is illustrated for the trauma film (left), the neutral film condition (middle), and the difference between the two conditions (right). None of the measured frequency bands showed significantly different activities between trauma and neutral film conditions.

**Corresponding to Results: Effects of experimental trauma film on arousal, mood, and sleep architecture**

**Figure S4. Heart rate during film presentations**

*Note.* Mean heart rate (bpm) across the 12-minute film viewing period for the trauma and neutral film conditions. Ribbons represent 95% confidence intervals of the mean. Significant differences in heart rate between conditions were observed during the initial minute (0–1), the emotionally intense segment (7–10), and the final segment (11–12), indicated by brackets and asterisks (* < .05, ** < .01, *** < .001).

**Corresponding to Results: Prediction of intrusions and affective response to trauma film reminders**

**Figure S5: Results on sleep spindle density**

*Note.* Topographical distribution of the mean power in spindle density by condition and correlations between intra-individual change (experimental minus neutral night) in spindle density and heart rate (HR), intrusions, and negative affect. No significant difference and no significant correlations were found.

**Corresponding to Methods: Intrusion Diary**

**Supplementary Material M1: Further information on the intrusion dairy protocol**

To support compliance, participants were instructed to document intrusions as closely in time to their occurrence as possible via a smartphone-based diary link. Additionally, we sent daily email reminders in the evening to encourage participants to reflect on and document any intrusions they may not have reported earlier in the day.

Importantly, we also conducted a structured debriefing with each participant after the six-day diary period. During this session, participants were asked to review their entries day-by-day with the experimenter. For each day, they were reminded of the intrusions they had documented and asked whether there were any additional intrusions that they had not recorded, and whether their entries reflected the most salient or all intrusive experiences. Participants were also asked whether the diary served as a cue for remembering intrusions (e.g., entering them retrospectively), or whether they primarily made spontaneous entries. If participants mentioned any additional intrusions during this debriefing, these were documented and added to the dataset.

**Corresponding to Methods: Sleep EEG recordings and analyses**

**Supplementary Material M2: Further information on spindle detection and quantification of slow wave activity**

Discrete sleep spindle events (12 - 16Hz) were detected during artifact-free intervals of NREM sleep 2 and 3 as described in (Ngo et al., 2020). In brief, the EEG signals were band-pass filtered between 12–16 Hz and the root mean square signal (RMS) was calculated based on a 200-ms window followed by an additional smoothing using a moving average with the same window length. A spindle event was identified whenever the smoothed RMS-signal exceeded a threshold, defined by the mean plus 1.2816 times the standard deviation (i.e., corresponding to the 90th percentile) of the RMS-signal across all NREM data points, for at least 0.4 seconds but not longer than 3 seconds. The upward and downward threshold crossings represent the beginning and end of a spindle event. The spindle envelope was determined by averaging the smoothed RMS-signal for the duration of each detected spindle event. Identical to the other spectral analyses, topographical maps based on the spindle count and spindle activity at each electrode averaged across participants were created separately for the neutral and trauma film condition. The difference between conditions was visualised in a topographical map after subtracting the corresponding values at each electrode and averaged across participants

Finally, to quantify the intra-individual increase in SWA (0.5–4 Hz) after sleep onset, EEG power was segmented into consecutive two-minute intervals during the first NREM sleep cycle. For each participant, the SWA power was computed at each electrode across all intervals. At every channel, the initial SWA value (i.e., power during the first two-minute segment) and the peak SWA value (i.e., highest power across the first cycle) were identified. The SWA slope was then calculated by dividing the difference between the peak and the initial value by the number of intervals elapsed until the peak was reached: SWA slope = (SWA_max − SWA_initial) / interval_max_position. This measure captures the rate of SWA buildup within the first sleep cycle and was computed separately for the trauma and neutral film conditions. This dynamic approach allows for assessing individual differences in early-night homeostatic sleep regulation, potentially relevant for emotional memory processing.
