# Supplementary figures and images for: Sleep to remember, sleep to protect: increased sleep spindle and theta activity predict fewer intrusive memories after analogue trauma
Source: Transl Psychiatry. 2026 Feb 17;16:147. doi: 10.1038/s41398-026-03910-0 (PMC12987997; doi:10.1038/s41398-026-03910-0)

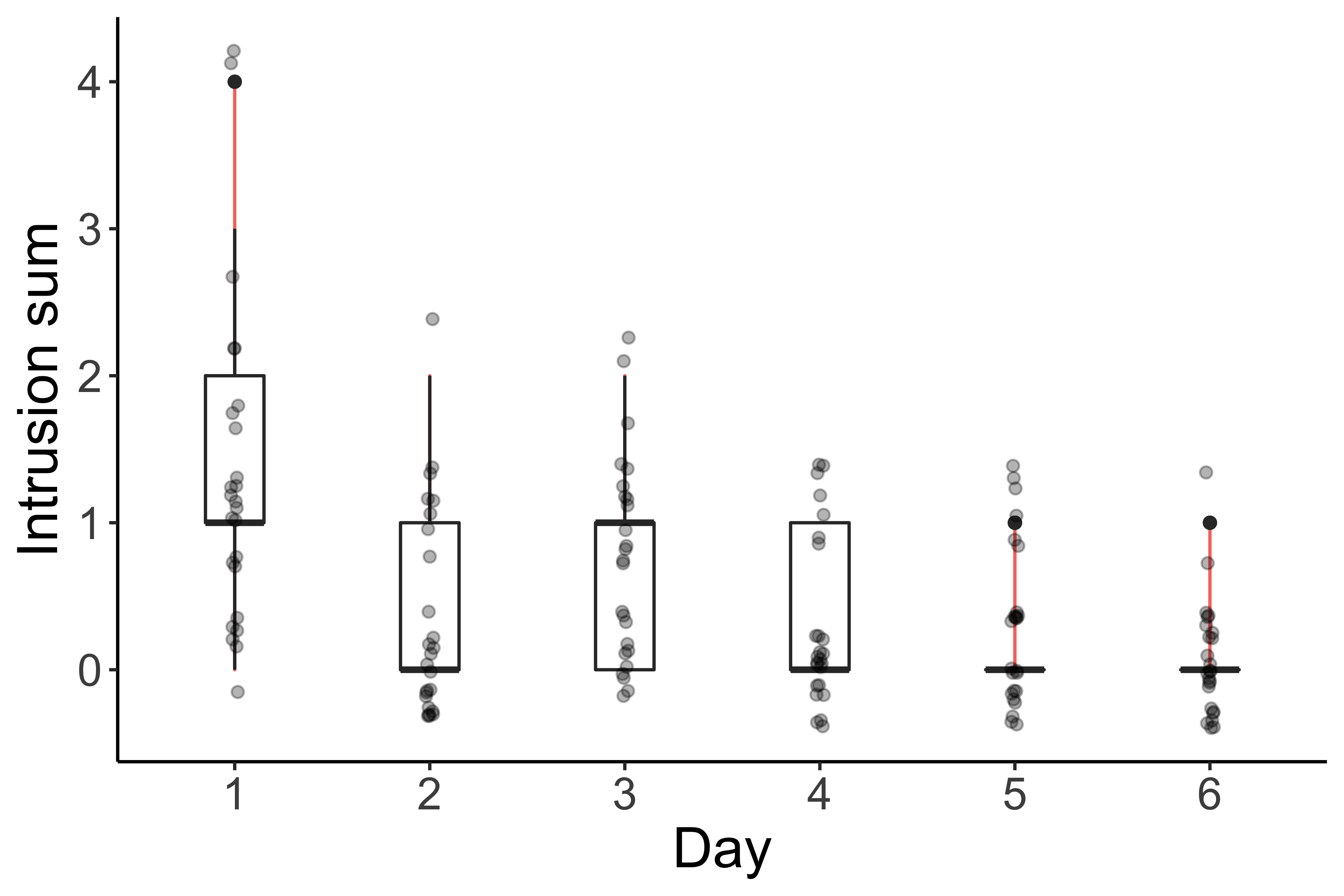

Supplement: Supplementary file 2 — Supplement Figure S1 [file 41398_2026_3910_MOESM2_ESM.png]

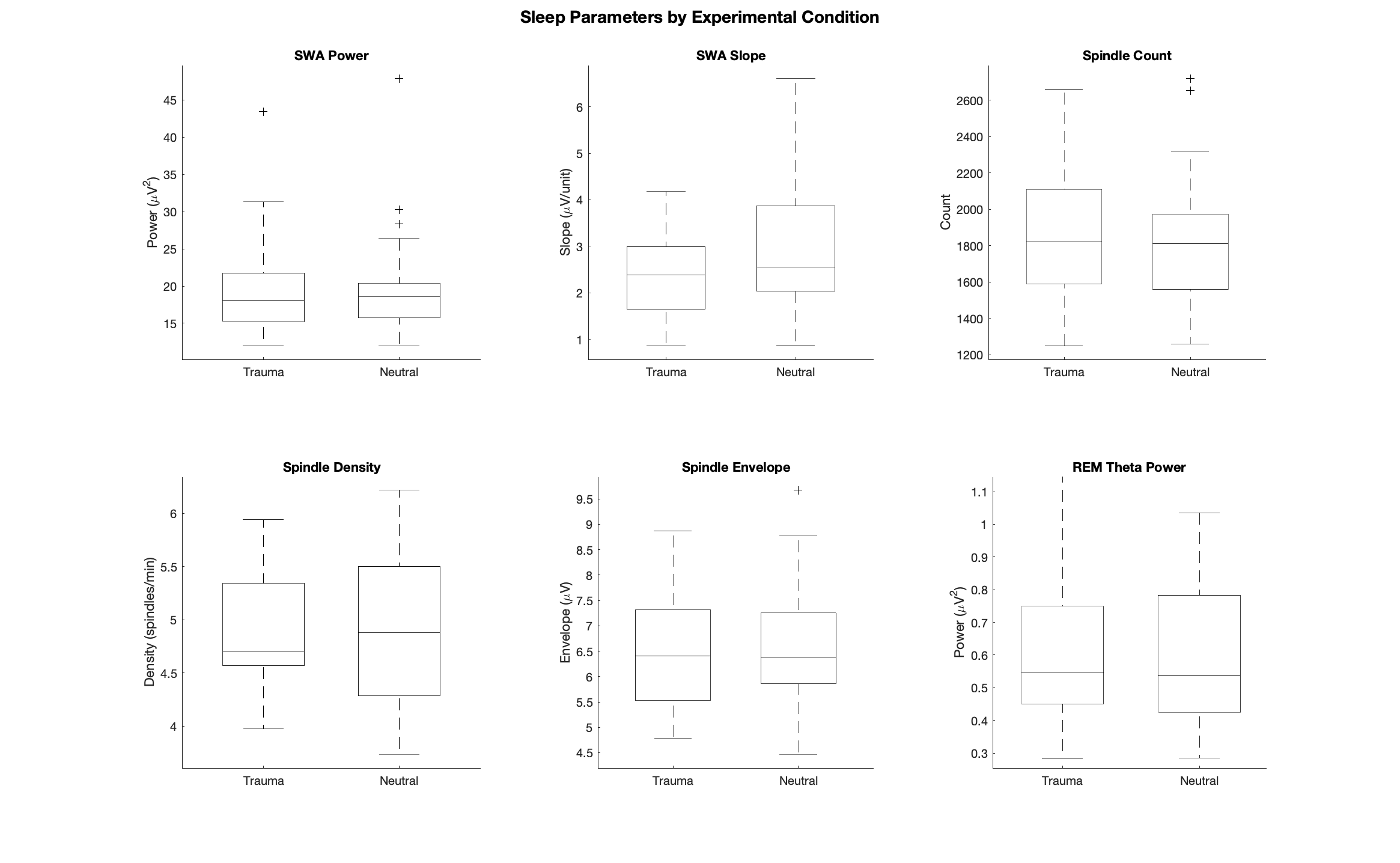

Supplement: Supplementary file 3 — Supplement Figure S2 [file 41398_2026_3910_MOESM3_ESM.png]

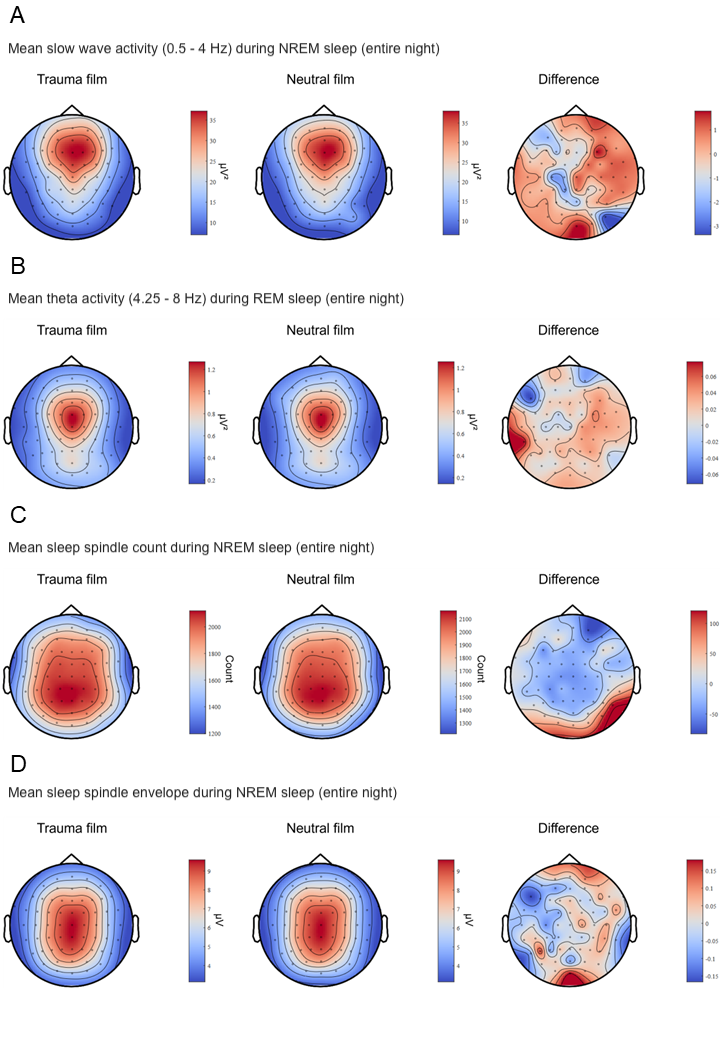

Supplement: Supplementary file 4 — Supplement Figure S3 [file 41398_2026_3910_MOESM4_ESM.png]

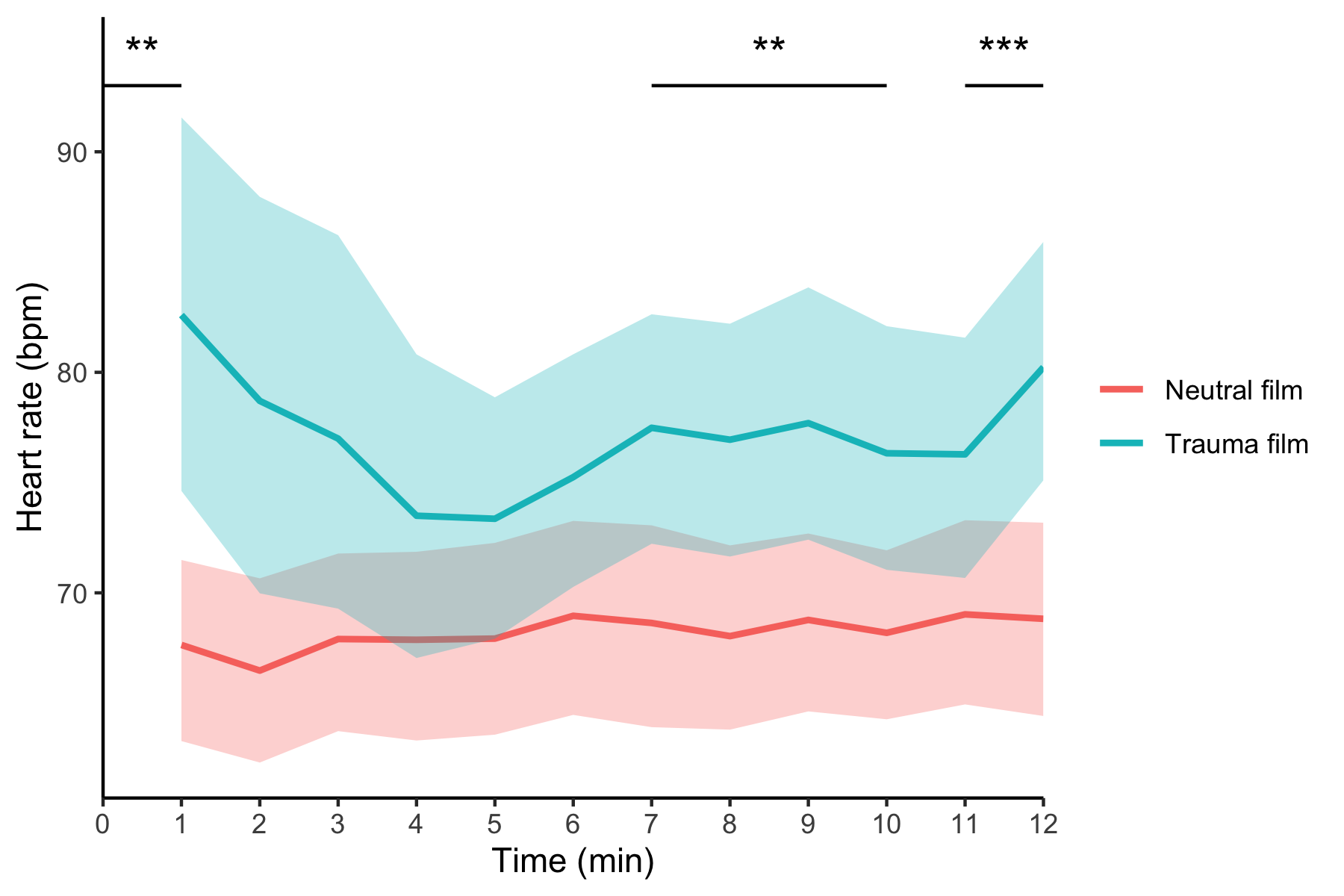

Supplement: Supplementary file 5 — Supplement Figure S4 [file 41398_2026_3910_MOESM5_ESM.png]

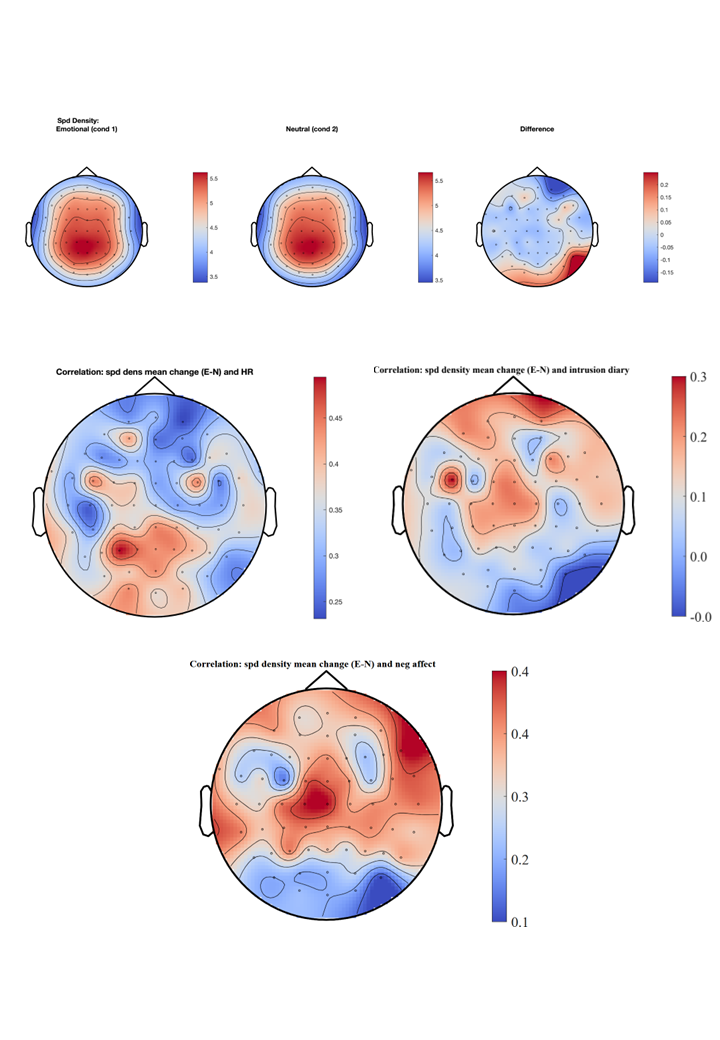

Supplement: Supplementary file 6 — Supplement Figure S5 [file 41398_2026_3910_MOESM6_ESM.png]
